# Subcellular distribution and chemical forms of manganese in *Daucus carota* in relation to its tolerance

**DOI:** 10.3389/fpls.2022.947882

**Published:** 2022-10-06

**Authors:** Xueshao Kuang, Wumin Wang, Jiayao Hu, Wensheng Liu, Wenbin Zeng

**Affiliations:** College of Life Science and Technology, Central South University of Forestry and Technology, Changsha, China

**Keywords:** *Daucus carota*, Mn accumulation, anatomical structure, subcellular distribution, chemical forms

## Abstract

*Daucus carota* is a biennial herb of the Umbelliferae family, which is a candidate plant for the phytoremediation of Mn pollution. To reveal the mechanism of this plant to adapt to Mn stress, plant growth, anatomical structure, Mn accumulation characteristic, Mn subcellular distribution, and chemical forms of *D. carota* under six Mn^2+^ concentrations by pot culture experiments were studied. The results showed that with the rising Mn concentrations, the total dry weight and leaf area of *D. carota* increased firstly and then decreased, while the specific leaf area increased. The thickness of the main vein, upper epidermis, and lower epidermis; the thickness of the palisade tissue; and the thickness of the spongy tissue of the leaves increased firstly and then decreased. The Mn content in the aboveground and underground parts of *D. carota* increased, and the values of the bioconcentration factor (BCF) and translocation factor (TF) were higher than 1. The Mn existing in the cell wall and soluble components accounted for the largest proportion, and the proportion of Mn in the cell wall increased with increasing concentrations of Mn. In addition, Mn mainly existed in ethanol extraction state, deionized water extraction state, and sodium chloride extraction state. The results showed that *D. carota* could alleviate the damage caused by high manganese concentration by storing most of manganese in the cell wall and vacuole and existing in the form of low-activity state.

## Introduction

Manganese (Mn) is one of the essential trace elements for plant growth, which is involved in photosynthesis, while overdose of Mn in plants would interfere with the absorption and utilization of other mineral elements, influence energy metabolism, depress photosynthetic rates, and lead to oxidative stress (Fernando & Lynch, 2015; Tavanti et al., 2020), which would inhibit plant growth and development and even endanger human health through the food chain (Yao et al., 2012). In the field ecosystems, human activities such as mining, metallurgy, and illegal sewage discharge lead to Mn pollution in soil. It was reported that manganese toxicity is one of the main toxic factors inhibiting the crop growth in acid soil in China, and it is an urgent task to control the pollution (Xu et al., 2009; Huang et al., 2019; Xiao et al., 2019). Among various controlling technologies, phytoremediation is the one with low cost, wide range of adaptation, small impact on the original environment, and long-lasting governance effect (Bhaduri and Fulekar, 2012; Gallego et al., 2012; Tavanti et al., 2020). The premise of this technology is to screen plants with high heavy metal tolerance and high economic value (Huang et al., 2018; Wu et al., 2018). Therefore, screening plants with tolerance to heavy metals and revealing their tolerance mechanism is the key step for the remediation of contaminated soil by phytoremediation technology.

Morphological and microstructure changes can directly and comprehensively reflect the resistance of plants to environmental stress (Daud et al., 2009). Under stress, plant morphology would make some protective compensation responses to adapt to gradual or sudden changes of the environment (Elleuch et al., 2013). It was reported that low concentrations of Mn treatment can stimulate the activity of certain enzymes and promote plant growth (Mou et al., 2011), and high concentrations of the Mn treatment would cause plant biomass reduction (Yao et al., 2012). For example, the dry weight of roots, stems, and leaves of *Macleaya cordata* under low Mn treatment was significantly higher than that of the control (0 μM of Mn) (Pan et al., 2019b). The root growth of *Broussonetia papyrifera* increased firstly and then decreased with the increasing Mn concentration (Huang et al., 2019). Leaves are the main organs of photosynthesis and transpiration for plants; its structural characteristics reflect the adaptation of plants to the environment (Tian et al., 2014). For example, under high concentrations of Mn stress, the thickness of leaf palisade tissue and sponge tissue of *Populus cathayana* decreased significantly, indicating that mesophyll was seriously damaged under Mn stress (Lei et al., 2007).

In heavy metal-contaminated soils, plants cope with the potential heavy metal stress in different ways. Some plant species adopt an exclusion strategy to avoid the excessive uptake and transport of metal ions. In contrast, other plant species can take up large amounts of metals by roots and transport them to the shoots. The difference in the absorption and accumulation of Mn by different plants reflects its strategy. For example, the Mn content in plant tissues *Phytolacca acinosa* (Xue et al., 2004), *Alyxia rubricalis* (Marques et al., 2009), and *Polygonum lapathifolium* (Liu et al., 2016) was high and increased with the increasing Mn treatment concentration.

Subcellular distribution and chemical forms of heavy metal were associated with metal tolerance and detoxification in plants. In order to survive in Mn-contaminated soil, plants have evolved various strategies for Mn detoxification, including metal exclusion, binding of Mn to the cell wall, restriction of Mn accumulation in sensitive tissues/organelles, and sequestration in vacuoles. At the cellular and tissue levels, heavy metals are mainly distributed in the cell wall and vacuole, isolating them from metabolically active areas such as cytoplasm, mitochondria, and chloroplasts, so as to maintain the smooth progress of normal physiological metabolism (Wang et al., 2015). For example, the Mn content of the cell wall and vacuole in *Phytolacca americana* (Dou et al., 2009) and *Vitis vinifera* (Yao et al., 2012) was the highest, which illustrated that compartmentalization plays an important role in tolerant plants (Li et al., 2018).

The chemical form approach quantifies the metal fates within cells by sequentially extracting metals with different chemical solutions (Jiang et al., 2021). Although these chemical phases are operationally defined, studies have provided clear evidence that heavy metals in inorganic form and water-soluble form migrate more readily than metals binding to pectates, phosphates, and oxalates, and these forms have stronger negative effects on plant cells. The organic compounds secreted by plants can not only activate the heavy metals in the soil but also form stable metal chelates with them, reduce their toxicity to plant tissues, and control the absorption and migration of heavy metals (Daud et al., 2009). Most of the heavy metal ions absorbed by plants will be transformed into different chemical forms, and the chemical form is directly related to the migration ability, activity, and toxicity of heavy metals in plants. For example, Wang et al. (2007) found that deionized water-extracted Mn is the main form of occurrence in the roots, stems, and leaves of *Polygonum hydropiper*, which is conducive to the transportation and accumulation of Mn to the aboveground parts of plants. In the mining area, Mn in the leaves, stems, and roots of *Xanthium strumarium* mainly exists in the form of pectinate and protein binding state (21.88%~55.43%), phosphate (9.21%~39.29%), and Mn oxalate (3.20%~22.19%), while heavy metals in the form of phosphate and oxalate are mainly stored in plant cell walls and vacuoles, and heavy metals combined with pectin and protein are mainly stored in vacuoles (Pan et al., 2019a). This may be one of the reasons that plants can resist high Mn stress.

*Daucus carota* is a biennial herb of carrot belonging to Umbelliferae. It has the characteristics of large biomass, beautiful plant type, wide ecological adaptability, fast growth rate, and strong stress resistance. A field investigation showed that this plant is widely distributed in Xiangtan Mn Mine Wasteland and can flower and bear fruit normally, indicating that the plant has strong Mn tolerance (Wu et al., 2019). At present, the related research of *D. carota* mainly focused on biological activity (Li et al., 2012) and plant response under cadmium and copper stress (Wu et al., 2006; Ke et al., 2007; Dong et al., 2009). However, there are few studies on the tolerance and mechanism of *D. carota* under Mn stress, which limited the application of this plant in Mn pollution control. In order to reveal the response and tolerance mechanism of *D. carota* to manganese stress, this study analyzes the growth characteristics, leaf anatomical structure, Mn accumulation ability, subcellular components, and chemical morphological characteristics of *D. carota* under Mn stress. The aim of this study is to address the following questions. (1) Are there variations in Mn accumulation under different Mn exposure conditions? (2) Are these variations associated with Mn subcellular distribution, chemical forms? The aim is to provide a theoretical basis for the application of *D. carota* in the remediation of a manganese-polluted environment. Characterization of mechanisms involved in Mn accumulation and detoxification will provide a basis for further screening or engineering Mn-tolerant plants.

## Materials and methods

### Seed collection

Field investigations showed that *D. carota* in the Xiangtan Mn Mine area of Hunan Province (112°85′E, 27°97′N) distributes widely, grows well, and sets many fruits in this area. In October 2019, about 100 *D. carota* plant individuals were randomly selected and mature fruits were collected and taken back to the laboratory by paper bag. After natural drying, they were stored at room temperature. The concentration of Mn in the soil in this area is about 52,319.25 mg·kg^-1^.

### Plant cultivation under controlled conditions

In May 2020, plump and uniform-size seeds were selected and sown in a sterilized sand dish and placed in an artificial climate incubator with a light intensity of 5,000 lx, a temperature of (25 ± 1)°C, a photoperiod of 12 h/12 h (day/night), and a relative humidity of 70%. When the seedlings grew to 4~6 cm in height, 1/2 concentration of Hoagland nutrient solution was applied. When the seedlings grew to 8~10 cm in height, seedlings with the same height were transplanted to the plastic pots (20 cm in diameter, 14 cm in height) containing fine sand and perlite (1:1). During the culture period, deionized water was added every day to maintain the growth substrate moist, and Hoagland nutrient solution was applied every 5 days. According to our preliminary experiments, the highest concentration of *D. carota* tolerant to Mn is 20,000 μmol·l^-1^. Therefore, according to the method of Pan et al. (2019), six Mn concentration treatments were set (0 (CK), 1,000, 5,000, 10,000, 15,000, 20,000 μmol·l^-1^) by using MnCl_2_·4H_2_O. After the seedlings were transplanted to the pots for 2 weeks, six concentrations of Mn solutions were added into the pots. Twenty pots per treatment were set, and 120 pots were set in total.

### Determination of growth indicators

On the 30th day after carrying out the Mn treatment, 10 individuals were randomly selected in each treatment. A vernier caliper with an accuracy of 0.1 mm was used to measure the plant height and taproot length, respectively. Then, the plants were washed with deionized water. After the plants were dried, the root morphology was measured with an Epson scanner (expression 11,000 xl, Japan). The root surface area, root volume, root diameter, and fibrous root number were scanned and analyzed by WinRHIZO image analysis software (2013e, Regent Instruments Inc., Canada). The leaf area was measured with the same method. Finally, the plant individuals were put in the oven, scalded at 95°C for 10 min, and baked at 65°C to constant weight, and the dry weight was weighed. The specific leaf area (SLA, the ratio of leaf area and leaf dry weight) was also calculated.

### Determination of leaf anatomical structure

After 30 days of Mn treatment, 20 mature leaves were selected from each treatment, and anatomical structures were studied by using the paraffin section method. The size of the cut was controlled to 5 mm × 5 mm and placed in FFA fixative solution (70% ethanol: formaldehyde: glacial acetic acid = 90:5:5), sealed and stored in a brown glass bottle, and stored at 4°C for later use. The fixed and preserved leaves were pumped again, and the leaves were selected for ethanol gradient dehydration (85% ethanol, 95% ethanol, and absolute ethanol), made transparent in xylene, and embedded after soaking in wax. A digital microscope (Motic Images Advanced 3.0) was used to observe and take pictures. Digitizer Image Measurement software was used to measure leaf thickness, upper epidermis thickness, lower epidermis thickness, palisade tissue thickness, sponge tissue thickness, and other related data. The ratio of palisade tissue thickness and spongy tissue thickness and the tightness of organizational structure (the ratio of palisade tissue thickness and blade thickness) were calculated.

### Determination of Mn content

The content of Mn in the aboveground and underground parts of *D. carota* was determined by atomic absorption spectrometry (Yao et al., 2012). After 30 days of stress, the whole plant was harvested and divided into two parts: the aboveground part and the underground part, cleaned with distilled water and dried to constant weight. After being ground and burned, the ash content was dissolved with HCl. Finally, the content of Mn was determined using an atomic absorption spectrophotometer (AA-7000, Shimadzu, Japan). The bioconcentration factor (BCF, the ratio of total Mn content of plant samples and matrix heavy metal content) and transport factor (TF, the ratio of aboveground Mn content and underground Mn content) were also calculated.

### Subcellular distribution of Mn

The subcellular components of Mn were determined by differential centrifugation according to the improved method of Pan et al. (2019). Three grams of fresh plant samples was chosen and mixed with 9 ml subcellular extract (0.25 mmol·l^-1^ sucrose, 50 mmol·l^-1^ Tris–HCl buffer (pH 7.5), 1 mmol·l^-1^ dithioerythritol) and was ground into homogenate on ice bath. Then, they were transferred to a centrifuge tube and centrifuged at 300 r·min^-1^ for 5 min. The sediment was the cell wall component (F1). Then, the supernatant was centrifuged at 2,000 r·min^-1^ for 20 min, and the sediment was chloroplast and cell nuclear components (F2). In addition, the supernatant was centrifuged at 10,000 r·min^-1^ for 20 min, the residue was the mitochondrial component (F3), and the supernatant was the ribosomal component (F4, soluble component). The obtained components were transferred to a porcelain crucible without damage and dried to constant weight at 70°C. Then, the mixed acid HNO_3_–HClO_4_ (5:3, v/v) was added on the hot plate and boiled until the residue is completely dissolved and becomes a powder. Finally, it was fully dissolved, rinsed, and filtered with deionized water; the volume in a 25-ml volumetric flask was fixed and diluted to an appropriate multiple; and an atomic absorption spectrophotometer (AA-7000, Shimadzu, Japan) was used to determine the Mn content in the sample.

### Determination of Mn chemical speciation

The chemical forms of Mn in plants were extracted using a sequence of different extractants as the method of Pan et al. (2019): (1) 80% ethanol, extracting inorganic Mn giving priority to nitrate/nitrite, chloride, and aminophenol manganese; (2) deionized water (d-H_2_O), extracting water-soluble Mn–organic acid complexes and Mn(H_2_PO_4_)_7_; (3) 1 M NaCl, extracting pectates and protein-integrated Mn; (4) 2% acetic acid (HAC), extracting undissolved manganese phosphate including Mn_2_(HPO_4_)_7_ and Mn_2_(PO4)_7_ and other Mn–phosphate complexes; (5) 0.6 M HCl, extracting manganese oxalate; (6) Mn in residues.

Three grams of fresh plant samples was taken, mixed with 20 ml extractant (80% ethanol, 1 mol·L^-1^ sodium chloride, 2% acetic acid, 0.6 mol·l^-1^ hydrochloric acid), and ground into a homogenate, then transferred to a centrifuge tube, shaken at 25°C for 22 h, and centrifuged at 5,000 r·min^-1^ for 10 min. After pouring out the supernatant, 10 ml of the same extractant was continuously added, shaken for 2 h, and centrifuged for 10 min, and the supernatant was combined. The supernatant was respectively inorganic Mn, water-soluble organic acid Mn, pectate and protein-bound Mn, Mn phosphate, and oxalate Mn, and finally precipitates as residual state. Various chemical forms were transferred into a porcelain crucible without damage and dried at 70°C to a constant weight. Then, HNO_3_–HCIO_4_ (3:1) mixed acid was added, heated, and digested on a hot plate until it became an off-white solid. Finally, 10% HNO_3_ was used to dissolve, wash, filter, and fix the volume, and the content of Mn was determined using an atomic absorption spectrophotometer.

### Statistical analysis

The data of each index were expressed as “mean ± standard deviation”. SPSS 19.0 statistical software was used to conduct one-way ANOVA on the measured data, and the least significant difference method (LSD method) was used to make multiple comparisons of the significance of the difference between different treatments for each indicator.

## Results

### Effect of Mn stress on plant biomass

With the increasing Mn concentrations, the dry weight of aboveground and underground parts of *D. carota* plants increased firstly and then decreased, reaching the maximum at 1,000 μM (**Table 1**), and the root-to-shoot ratio decreased. The aboveground height, aboveground dry weight, underground length, underground dry weight, and root-to-shoot ratio reached the highest value at 1,000 μM and reached the lowest value at 20,000 μM. Compared with the control, these values at 1,000 μM increased by 20.35%, 8.36%, 22.29%, 36.77%, and 11.76%, and reduced by 27.89%, 60.23%, 62.14%, 82.06%, and 52.94%, respectively.

**Table 1:** Effects of manganese stress on plant growth of *D. carota.*

Treatment (μmol·L-1)	Aboveground height (cm)	Underground length (cm)	Aboveground dry weight (mg/plant)	Underground dry weight (mg/plant)	Root-shoot ratio
0	32.23±2.65 b	30.85±1.51 a	6.55±0.73 b	2.23±0.20 b	0.34±0.05 ab
1000	38.79±1.58 a	33.43±1.98 a	8.01±0.34 a	3.05±0.39 a	0.38±0.06 a
5000	30.57±1.84 b	26.86±2.13 b	5.86±0.21 c	1.74±0.30 c	0.30±0.04 b
10000	27.30±0.91 c	22.23±1.29 c	3.78±0.16 d	0.94±0.08 d	0.25±0.03 b
15000	25.37±1.16 cd	16.73±1.71 d	2.87±0.22 e	0.58±0.02 de	0.20±0.01 bc
20000	23.24±1.42 d	12.27±0.92 e	2.48±0.27 e	0.40±0.01 e	0.16±0.02 c

Values are the mean ± S.D. (n=5). Values with different letters within the same column indicate significant differences at the P < 0.05 level between concentrations according to LSD test. The same as below.

As the concentration of Mn increased, the root surface area, root volume, average root diameter, and fibrous root number of *D. carota* increased firstly and then decreased (**Table 2**). All of these values (the root surface area, root volume, average root diameter, and fibrous root number) reached the maximum under 1,000 μM Mn treatment, and compared with the control, it increased by 31.56%, 14.40%, 27.64%, and 31.70%, respectively (**Table 2**).

**Table 2:** Effects of manganese stress on root characteristics of *D. carota.*

Treatment (μmol·L-1)	Root surface area (cm^2^)	Root volume (cm^3^)	Average root diameter (mm)	Root tip number
0	61.53±2.22 b	0.89±0.07 b	0.48±0.02 b	1164.00±75.44 b
1000	80.94±5.63 a	1.14±0.02 a	0.55±0.02 a	1533.00±81.66 a
5000	53.22±1.44 c	0.70±0.02 c	0.43±0.04 c	1093.67±62.04 b
10000	32.11±2.43 d	0.46±0.03 d	0.36±0.02 d	744.67±50.00 c
15000	16.49±1.15 e	0.29±0.02 de	0.33±0.01 e	331.33±20.84 d
20000	12.45±0.48 e	0.17±0.01 e	0.30±0.01 f	126.33±8.33 e

**Figure 1:**
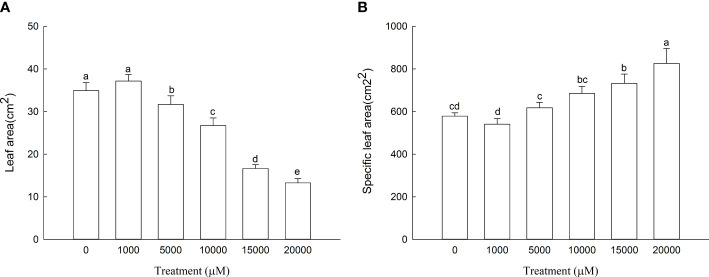
Leaf area **(A)** and specific leaf area **(B)** of *D carota* under Mn stress. Data points and error bars represent mean and S.D. (n = 3). Values with different letters indicate significant differences at the *P* < 0.05 level between different Mn concentrations according to the LSD test.

The leaf area of *D. carota* increased firstly and then decreased with the increase in Mn treatment concentration (**Figure 1A**), and the leaf area of 1,000 μM of Mn treatment reached the maximum. As shown in **Figure 1B**, the specific leaf area of *D. carota* generally showed an upward trend with the increase in concentration.

### Anatomical structure

**Figure 2:**
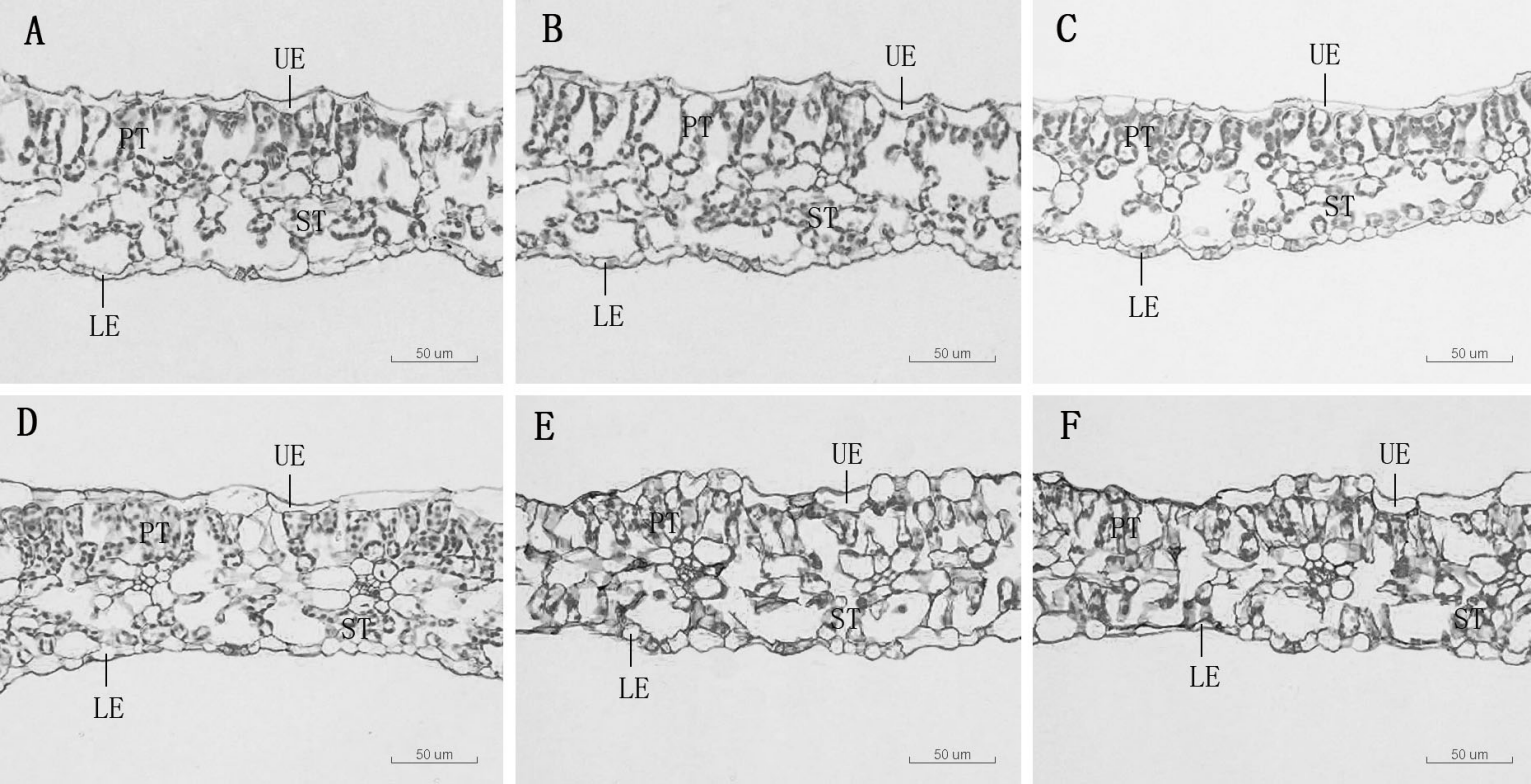
Leaf transverse anatomical structure of *D. carota* under Mn treatments **(A–F)**, UE, upper epidermis, LE, lower epidermis, PT, palisade tissue, ST, spongy tissue. **(A)** mdash;CK, **(B) **1,000 µM, **(C)** 5,000 µM, **(D) **10,000 µM, **(E)** 15,000 µM, **(F)** 20,000 µM.

As Mn concentrations increased, the thickness of the main vein, the thickness of the upper epidermis, and the thickness of the lower epidermis of *D. carota* increased firstly and then decreased, while the leaf thickness decreased (**Table 3**; **Figure 2**). The thickness of the upper and lower epidermis and the main vein reached the maximum value under the treatment of 5,000 µM of Mn, and the minimum value was reached at 20,000 µM.

**Table 3:** The effects of Mn treatments on epidermal tissue and main vein structure in *D. carota*.

Treatment (μmol·L-1)	TE (μm )	TUE (μm )	TLE (μm )	TMV (μm )
0	119.79±1.76 a	10.50±0.76 bc	7.72±0.67 ab	169.24±10.04 ab
1000	106.20±2.32 b	11.25±0.65 b	7.44±0.57 b	162.37±3.57 b
5000	102.45±4.30 b	13.76±0.26 a	8.64±0.69 a	174.47±4.51 a
10000	100.40±4.58 b	8.75±0.14 c	7.33±0.52 c	149.24±3.16 c
15000	90.31±2.44 c	7.99±0.85 c	6.86±0.12 c	145.37±3.49 c
20000	95.75±7.35 c	7.21±0.32 c	6.63±0.26 c	142.54±3.06 c

TE, the leaf thickness; TUE, the thickness of the upper epidermis; TLE, the thickness of the lower epidermis; TMV, the thickness of the main vein.

**Figure 3:**
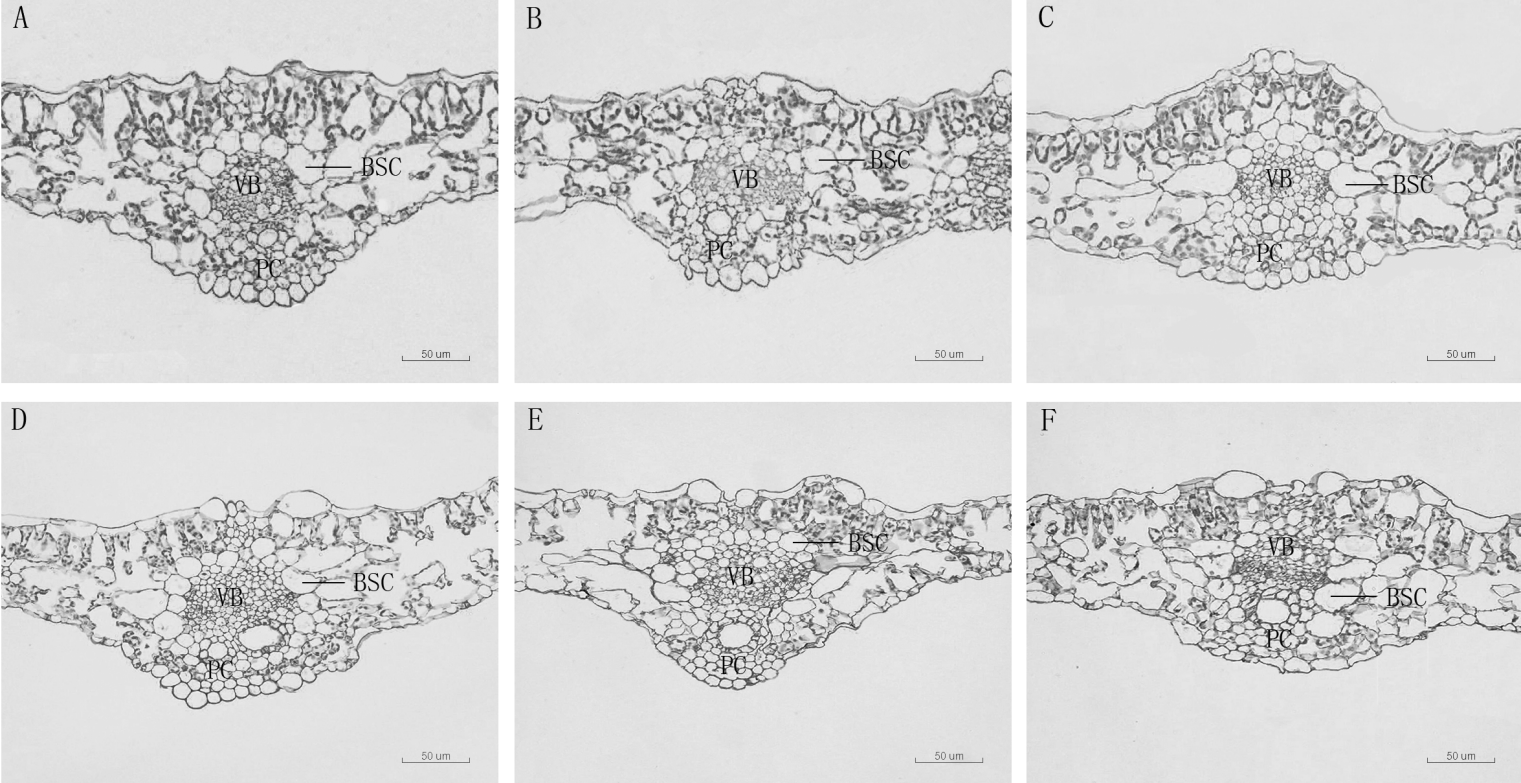
Leaf vein transverse anatomical structure of leaf blade of *D. carota* under Mn treatments **(A–F)**. BSC: bundle sheath cells, V, vascular bundle, C, pachycorn cells. **(A)** CK, **(B)** 1,000 µM, **(C)** 5,000 µM, **(D)** 10,000 µM, **(E)** 15,000 µM, **(F)** 20,000 µM.

The thickness of sponge tissue reached the maximum at 5,000 μM and then decreased with the increase in Mn stress concentration. The thickness and tightness of the tissue structure of the *D. carota* fence reached the maximum value at 1,000 μM Mn treatment then decreased with the increase in Mn concentrations (**Table 4**; **Figure 3**).

**Table 4:** The effects of excess Mn treatments on mesophyll tissue structure in *D. carota*.

Treatment (μmol·L-1)	TP (μm )	TS (μm )	RPS	CTR
0	35.01±2.77 b	37.55±0.30 c	0.93±0.07 a	0.29±0.02 bc
1000	45.49±6.83 a	68.13±10.23 ab	0.69±0.19 b	0.43±0.06 a
5000	39.43±1.43 ab	69.20±5.43 a	0.57±0.06 b	0.38±0.03 ab
10000	32.30±2.92 b	59.43±5.05 b	0.55±0.10 bc	0.32±0.03 b
15000	21.09±2.21 c	56.33±3.46 b	0.38±0.05 c	0.23±0.02 c
20000	16.85±2.33 c	45.37±2.65 c	0.37±0.05 c	0.18±0.04 c

TP, the thickness of palisade tissue; TS, The thickness of sponge tissue; RPS, The ratio of palisade tissue/ spongy tissue; CTR, cell tense ratio.

### Mn accumulation

**Figure 4:**
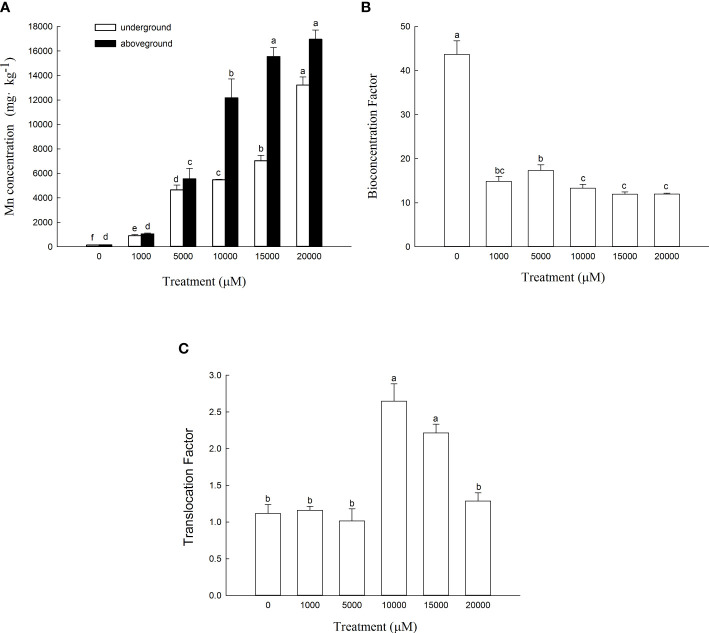
Mn concentrations in aboveground and underground parts **(A)**, bioconcentration factor (BCF) **(B)** and translocation factor (TF) **(C)** of D. carota under Mn stress. Data points and error bars represent mean and S.D. (n = 3). Values with different letters in the same parts of the plant indicate significant differences at the p < 0.05 level between different Mn concentrations according to the LSD test.

The Mn content in the aboveground and underground parts of *D. carota* increased with the increase in Mn concentration (**Figure 4A**). The content of the aboveground and underground parts of *D. carota* showed a linear cumulative trend with the rising Mn concentrations (for aboveground parts: y = 0.8993x + 929.49, *R*² = 0.9574; for underground parts: y = 0.5716x + 379.15, *R*² = 0.9276). When the Mn concentration attained to 20,000 µM, both the Mn content in the aboveground part and underground part reached the maximum, which were 16,948.37 and 13,210.33 mg·kg^-1^, respectively. The Mn content in the aboveground part of *D. carota* was higher than that in the underground part under different concentrations of Mn treatment. When the Mn treatment concentration ranged in 10,000~2,000 μM, the Mn content of the aboveground part of *D. carota* can reach over 10,000 mg·kg^-1^.

As the Mn concentration increased, the bioconcentration factor of *D. carota* decreased, and the least value was 11.92 (**Figure 4B**). The transport coefficients (TF) increased firstly and then decreased, and all of the values were greater than 1. When the Mn concentration was 10,000 μM, the transport coefficient was the highest, which was 2.65 (**Figure 4C**).

### Subcellular distribution of Mn

**Figure 5:**
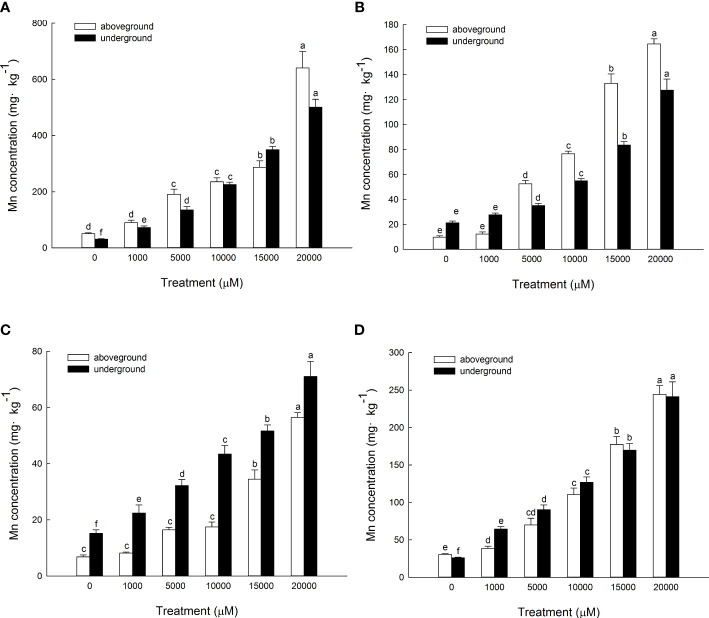
Concentrations of Mn in different subcellular compartments in *D carota* aboveground and underground under Mn stress (mg kg^-1^ FW) [**(A)** cell wall; **(B)** chloroplasts and cell nuclei; **(C)** mitochondria; **(D)** ribosomes)]. Data points and error bars represent mean and S.D. (n = 3). Values with different letters in the same parts of the plant indicate significant differences at the p < 0.05 level between different Mn concentrations according to the LSD test.

**Figure 6:**
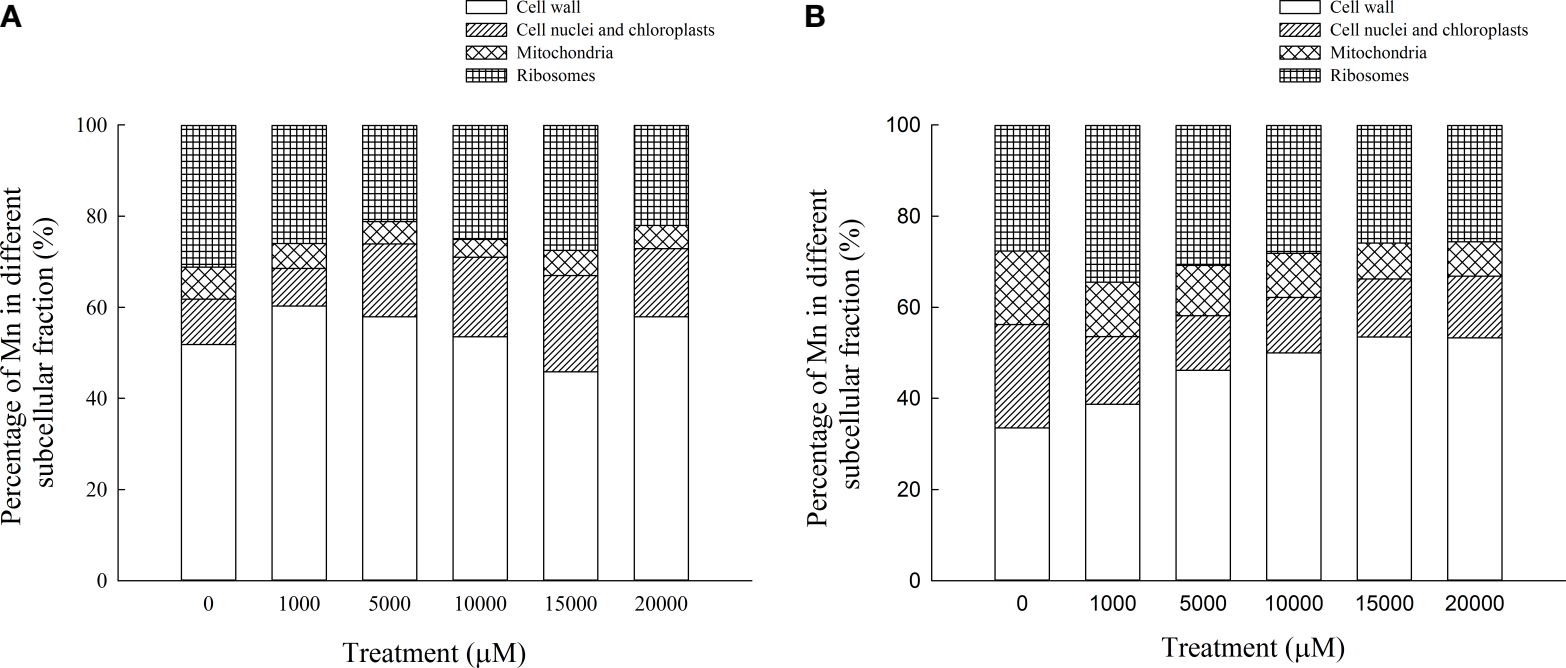
Percentages of subcellular distribution of Mn in aboveground part **(A)** and underground part **(B)** of *D carota* under different Mn treatments.

The content of Mn in cell wall components, chloroplast and nucleus components, mitochondrial components, and ribosome components of the aboveground part of *D. carota* increased with the increase in Mn treatment concentration (**Figures 5A–D**); the correlation coefficients were 0.862, 0.991, 0.912, and 0.985, respectively (*P* < 0.05). In the subcellular components of the aboveground part of *D. carota*, the cell wall component has the highest Mn content, followed by ribosomal component, chloroplast and nuclear component, and mitochondrial component; the contents of Mn in the cell wall and ribosome components accounted for 73.38%~86.24% of the total. Under the treatment of 20,000 μM Mn, the Mn content of each subcellular component reached the highest value; the values were 640.51, 164.59, 56.43, and 244.12 mg·kg^-1^, respectively (**Figure 6A**).

The content of Mn in cell wall components, chloroplast and nucleus components, mitochondrial components, and ribosome components in the underground part of *D. carota* increased with the increase in Mn treatment concentration; the correlation coefficients were 0.987, 0.950, 0.982, and 0.974, respectively (*P* < 0.05, **Figures 5A–D**). Among the subcellular components in the underground part of *D. carota*, the cell wall component has the highest Mn concentration, followed by the ribosomal component, chloroplast and nucleus component, and mitochondrial component which has the lowest content. The Mn content of cell wall components, chloroplast and cell nuclear components, ribosomal components, and ribosomal components all reached the maximum under the treatment of 20,000 μM Mn; they were 501.10, 127.56, 71.06, and 241.264 mg·kg^-1^, respectively (**Figure 6B**).

### Chemical forms of Mn

**Figure 7:**
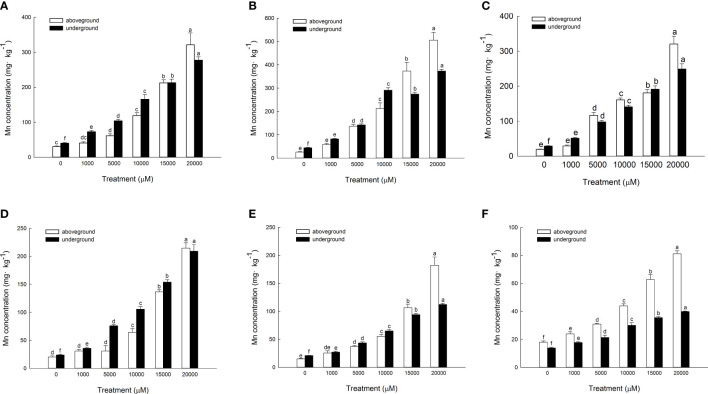
Concentrations of different Mn chemical forms in *D carota* aboveground and underground under Mn stress (mg kg^-1^ FW) [**(A)** 80% ethanol; **(B)** deionized H_2_O; **(C)** 1 M NaCl; **(D)** 2% HAc; **(E)** 0.6 M HCl; **(F)** residue]. Data points and error bars represent mean and S.D. (n = 3). Values with different letters in the same parts of the plant indicate significant differences at the p < 0.05 level between different Mn concentrations according to the LSD test.

**Figure 8:**
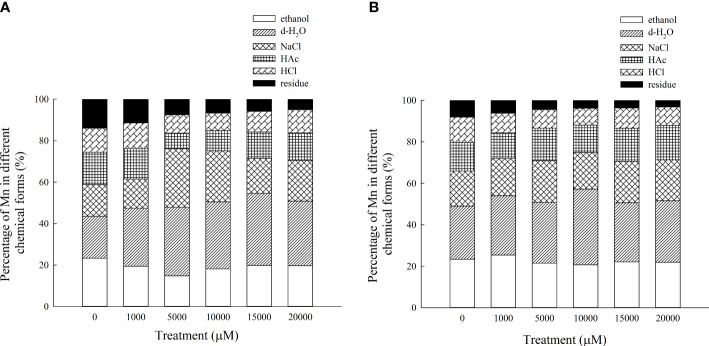
Percentages of different Mn chemical forms in aboveground part **(A)** and underground part **(B)** of *D carota* under different Mn treatments.

The Mn content in each chemical form of the aboveground part of *D. carota* is positively correlated with the Mn concentration; the correlation coefficients of ethanol, deionized water, sodium chloride, acetic acid, hydrochloric acid, and residual extracted Mn were 0.955, 0.987, 0.943, 0.910, 0.912, and 0.987, respectively (*P* < 0.05, **Figures 7A–F**). Its Mn content reached the maximum under 20,000 μM Mn treatment, which are 321.40, 505.98, 320.62, 214.67, 182.34, and 81.14 mg·kg^-1^, respectively. The proportion distribution trend of the chemical forms of Mn in the aerial parts is deionized water extraction state > sodium chloride extraction state > ethanol extraction state > acetic acid extraction state > hydrochloric acid extraction state > residual state (**Figure 8A**).

The Mn content in each chemical form in the underground part of *D. carota* is positively correlated with the Mn concentration; the correlation coefficients of ethanol, deionized water, sodium chloride, acetic acid, hydrochloric acid, and residual extracted Mn were 0.992, 0.931, 0.996, 0.993, 0.997, and 0.982, respectively (*P* < 0.05, **Figures 6A–F**). Its Mn content reached the maximum under 20,000 μM of Mn treatment; the values were 277.38, 373.40, 249.03, 209.17, 112.10, and 39.87 mg·kg^-1^, respectively. The content of Mn in the underground part of *D. carota* was lower than that in the aboveground part. The proportion distribution trend of the chemical forms of Mn in the underground part is deionized water extraction state > ethanol extraction state > sodium chloride extraction state > acetic acid extraction state > hydrochloric acid extraction state > residual state (**Figure 8B**).

## Discussion

The present study showed that *D. carota* can live normally under a high Mn concentration environment. This plant had high Mn accumulation ability. The content of Mn in cell wall components and soluble components of *D. carota* was the largest part. Mn mainly existed in the form of ethanol extraction, deionized water extraction, and sodium chloride extraction.

### Effects of Mn stress on plant growth

Biomass reflects the growth status of plants and is an important index to determine plant tolerance and the potential for phytoremediation (Xu et al., 2018). In this study, the dry weight of the aboveground part and underground part of *D. carota* increased firstly and then decreased with the increase in Mn concentration. This is consistent with the results of that of *Dianthus carthusianorum* (Załecka and Wierzbicka, 2002) and *Medicago sativa* (Cao et al., 2019) under Mn stress. It showed that low promotion and high suppression are a common phenomenon for Mn to the growth of plants.

The root system is the first organ that is exposed to environmental stress, and plants can adapt to the stress by adjusting the growth and metabolism of the root system. Because the root surface area, root volume, average root diameter, and number of fibrous roots are all indicators that reflect the growth and development of plant roots, it is important to study these indicators. This study showed that under the treatment of 1,000 μM of Mn concentration, the root surface area, root area, average root diameter, and number of fibrous roots of *D. carota* were all significantly higher than other treatments and the control, which illustrated that low concentrations of Mn can promote plant roots. This is consistent with the findings of Liu et al. (2010) on *Polygonum perfoliatum*.

Leaves are important organs for plants to photosynthesize and produce organic matter. The reduction in leaf area will directly affect the accumulation of plant biomass. The specific leaf area can reflect the ability of plants to obtain environmental resources; the smaller the specific leaf area, the stronger the plant’s ability to obtain water, nutrients, light, and carbon (Donovan et al., 2011). The present study showed that with the increase in Mn concentration, the leaf area of *D. carota* increased first and then decreased; this is consistent with the results of Xiao et al. (2020), which showed that the phenomenon of low leaf area and high inhibition is common. The specific leaf area of *D. carota* increased with the increase in Mn concentration; it is consistent with the results of Xiao et al. (2020) on *Cleome viscosa* under Mn stress and Wang et al. (2016) on *Cinnamomum camphora* under NaCl stress. The results showed that the ability of *D. carota* to obtain resources became weaker with the increase in Mn stress.

### Effects of Mn stress on anatomical structure

The leaf anatomical structure is closely related to the adaptability of plants to the stress environment. In this study, under a high concentration of Mn stress, the thickness of the leaf epidermis and the main vein of *D. carota* decreased, the palisade tissue became thinner, the sponge tissue became thicker, and the ratio of palisade tissue/spongy tissue decreased. It showed that under Mn treatment, plants will change their key morphological characteristics in order to adapt to adverse stress. This is consistent with the results of Lei et al. (2007) and Zambrosi et al. (2016), which illustrated that high concentrations of Mn stress reduced the thickness of palisade tissue and sponge tissue of the leaves of *Populus cathayana* and *Saccharum officinarum*. The reason lies in the high concentration of Mn hurt the plants, which would cause leaves to lose water and become thinner. The inhibition to plant growth when Mn reaches the threshold for toxicity in leaves is associated with the occurrence of oxidative stress due to an increased production of reactive oxygen species (ROS) in leaf tissues (Zambrosi et al., 2016).

### Effects of Mn accumulation characteristics

Mn accumulation ability reflects the strategy for plants to adapt to Mn stress; it is also the important index for screening phytoremediation plants. Normal concentrations of manganese in plant dry matter fall within the range of 20–500 mg/g and occasionally exceed 1,000 mg/g in plants on normal soils (Reeves and Baker, 2000). In this study, the maximum value of Mn concentrations in plants is 16,948 mg kg^-1^. It indicated that *D. carota* has a strong accumulation ability on Mn. The enrichment coefficient (BCF) and transport coefficient (TF) can reflect the enrichment capacity of plants for heavy metals, and the BCF and TF of *D. carota* were both greater than 1; it showed that *D. carota* has high Mn absorption and transport ability under laboratory conditions. Compared with other plants, such as *Macleaya cordata* (Pan et al., 2019b) and *Broussonetia papyrifera* (Huang et al., 2019), *D. carota* had higher values of BCF and TF, which further illustrated that *D. carota* had a great application potential to phytoremediation of soil environment by Mn pollution.

### Subcellular distribution of Mn

The subcellular distribution of heavy metals has important effects on the accumulation, migration, and detoxification of heavy metals in plants (Qiu et al., 2011). Plants would endure the toxicity of heavy metals through cell compartmentalization, bind excess metal ions to the cell wall, or separate them in vacuoles (Gallego et al., 2012). In this study, the percentage of Mn in the cell wall components of the aboveground and underground parts of *D. carota* was the largest, which accounted for 45.83%~60.26% of the total. This is consistent with the results of *Polygonum hydropiper* (Wang et al., 2007) and *Cleome viscosa* (Xiao et al., 2020). The cell wall was the first barrier against metals in plant cells and could bind a large number of metal ions and avoid transmembrane transport and migration of these ions into cells, thereby reducing the concentration of metal ions in the protoplasm, protecting plants from poison. The cell wall, which is mainly composed of cellulose, hemicellulose, lignin, pectin, a large number of functional proteins, and a small number of aromatic compounds, can store metal ions and prevent them from being transported across the cytomembrane.

The results of this study showed that the contents of Mn in the cell wall and ribosome components accounted for 73.38%~86.24% of the total, which illustrated that the soluble components of *D. carota* contained much more Mn than the chloroplast, nuclear components, and mitochondrial components. This is consistent with the results of *Xanthium strumarium* (Pan et al., 2019a) and *Polygonum hydropiper* (Wang et al., 2008). The reasons are that 95% of the volume of mature cells are vacuoles and the soluble components of cells are mainly composed of vacuoles. Therefore, the vacuole is the second largest place for storing Mn in *D. carotas* besides the cell wall. When the amount of heavy metals stored in the cell wall reached saturation, they would enter the protoplast through the cell membrane; most of them were transported to vacuoles and bound to their compounds, and heavy metal ions undergo fixation through compartmentalization, avoiding excessive accumulation of heavy metals in cell membranes and organelles, thereby protecting the cytoplasm from damage. The soluble fraction, which is mainly composed of vacuoles, acts as the secondary site of preferential Mn binding in the plant (Guan et al., 2018). Heavy metal ions would be transported to vacuoles when the cell wall becomes overwhelmed. Thus, vacuoles can act as a main “sequestration zone” to accumulate excess Mn in spinaches and provide further protection against toxicity for organelles.

Subcellular localization can help with the understanding of the mechanisms of heavy metal accumulation, transport, and detoxification in plants. After uptake by the plants, heavy metal detoxification is achieved by chelation and sequestration by organo-ligands at a subcellular level. The function of vacuoles in metal compartmentalization and the affinity of cell walls for heavy metals play an important role in metal detoxification and tolerance of plants.

### Chemical forms of Mn

The chemical form of Mn is one of the most important mechanisms to understand the accumulation and detoxification of Mn for plants (Lu et al., 2013), because it directly reflected the activity, toxicity, and migration ability of Mn. Mn in inorganic forms (extracted with ethanol) and in water-soluble forms (extracted with H_2_O) had the highest activity, followed by pectates and protein-bound Mn (extracted with NaCl) and undissolved Mn phosphates (extracted with HAc), and the Mn oxalate (extracted with HCl) and residues had the lowest activity. The migration ability and toxicity of Mn extracted by ethanol and deionized water (mainly in the free state or in the form of heavy metals combined with organic acids and water-soluble substances) are much stronger than those extracted by sodium chloride (mainly heavy metals combined with pectin and protein), acetic acid, and hydrochloric acid (mainly phosphate and oxalic acid heavy metals with low solubility). In this study, Mn in the aboveground part of *D. carota* mainly existed in the form of deionized water, sodium chloride, and ethanol extract. Moreover, Mn in the underground part mainly existed in the form of deionized water, ethanol extract, and sodium chloride. Mn in *D. carota* plants mainly existed in the form of water-soluble organic acid salts; it is conducive to the detoxification and upward transportation of Mn. Most of the heavy metals combined with pectin and protein are mainly isolated in vacuoles; it further showed that the aboveground part of *D. carota* detoxifies mainly by forming soluble Mn organic acid salts and accumulating in vacuoles. The same phenomenon also appeared in the study of *Polygonum hydropiper*, and *Phytolacca acinosa* (Xue et al., 2011) under Mn stress (Wang et al., 2008). With the increase in Mn concentration, the content of Mn oxalate in *D. carota* plants did not change significantly; it showed that oxalic acid accumulation may be a common characteristic of *D. carota*, which illustrated that oxalic acid was the main organic acid for detoxification of Mn accumulation in plant leaves. Different chemical forms of heavy metals are closely related to different biological functions, which have distinct bioavailability and toxicities. Water-soluble Mn in the inorganic form (extracted by 80% ethanol) and the organic form (extracted by deionized water) migrate more easily and are more toxic to plant cells than pectate and protein-integrated Mn (1 M NaCl extractable Mn), insoluble Mn phosphate complexes (2% HAC extractable Mn), and Mn oxalate (0.6 M HCl extractable Mn) with little or no toxicity to plants.

## Conclusions

In summary, the present study indicated that low concentrations of Mn treatment (0~1,000 μM) can promote the growth of *D. carota*, while high concentrations of Mn treatment (above 1,000 μM) can inhibit the growth. The Mn content in the aboveground and underground parts of *D. carota* were high and increased with the rising Mn concentrations. The content of Mn in cell wall components and soluble components was the largest part. In terms of chemical form, Mn mainly existed in the form of ethanol extraction, deionized water extraction, and sodium chloride extraction. It is concluded that Mn existed in the cell wall and soluble components as water-soluble organic acids and Mn oxalate, which might be an important Mn tolerance and detoxification mechanism for this plant. This study is still at a preliminary stage; further studies are needed to examine Mn storage sites and Mn abundance in the cells of plant tissue using promising techniques such as scanning electron microscopy (SEM) and energy-dispersive X-ray fluorescence spectrometry (EDX).

## Data availability statement

The original contributions presented in the study are included in the article/supplementary material. Further inquiries can be directed to the corresponding authors.

## Author contributions

WL designed this research. XK, WW and WZ analysed the data and wrote the manuscript. XK and JH performed the pot culture experiments. All authors contributed to the article and approved the submitted version.

## Funding

This study is supported by the National Natural Science Foundation of China (grant number 42177018) and the Hunan Provincial Natural Science Fund (2021JJ31147).

## Conflict of interest

The authors declare that the research was conducted in the absence of any commercial or financial relationships that could be construed as a potential conflict of interest.

## Publisher’s note

All claims expressed in this article are solely those of the authors and do not necessarily represent those of their affiliated organizations, or those of the publisher, the editors and the reviewers. Any product that may be evaluated in this article, or claim that may be made by its manufacturer, is not guaranteed or endorsed by the publisher.
